# The feasibility of conducting acute sarcopenia research in hospitalised older patients: a prospective cohort study

**DOI:** 10.1007/s41999-021-00565-6

**Published:** 2021-10-05

**Authors:** Carly Welch, Carolyn Greig, Zeinab Majid, Tahir Masud, Hannah Moorey, Thomas Pinkney, Thomas Jackson

**Affiliations:** 1 Medical Research Council (MRC)-Versus Arthritis Centre for Musculoskeletal Ageing Research, University of Birmingham and University of Nottingham, Birmingham and Nottingham, UK; 2 Institute of Inflammation and Ageing, College of Medical and Dental Sciences, University of Birmingham, Birmingham and Nottingham, B152TT UK; 3 University Hospitals Birmingham NHS Foundation Trust, Birmingham, B152GW UK; 4 School of Sport, Exercise, and Rehabilitation Sciences, University of Birmingham, Birmingham, B152TT UK; 5 Birmingham Biomedical Research Centre, University of Birmingham and University Hospitals Birmingham NHS Foundation Trust, Birmingham, UK; 6 University of Nottingham, Nottingham, UK; 7 Nottingham University Hospitals NHS Trust, Nottingham, UK; 8 Academic Department of Surgery, University of Birmingham, Birmingham, B152TT UK

**Keywords:** Acute sarcopenia, Feasibility, Frailty, Ultrasound, Bioelectrical impedance analysis

## Abstract

**Purpose:**

To assess feasibility of conducting acute sarcopenia research in complex populations of hospitalised older adults.

**Methods:**

Patients ≥ 70 years old were recruited to three cohorts: elective colorectal surgery, emergency (abdominal) surgery, medical patients with infections. Participants were recruited to the elective cohort in preoperative assessment clinic, and acutely admitted participants from surgical and medical wards at the Queen Elizabeth Hospital Birmingham. Serial measures of muscle quantity (ultrasound quadriceps, bioelectrical impedance analysis), muscle function (hand grip strength, physical performance), and questionnaires (mini-nutritional assessment, physical function) were performed at baseline, within 7 (± 2) days of admission/surgery, and 13 (± 1) weeks post-admission/surgery. Feasibility outcomes were assessed across timepoints including recruitment and drop-out rates, and procedure completion rates.

**Results:**

Eighty-one participants were recruited (mean age 79, 38.3% females). Recruitment rates were higher in elective (75%, 24/32) compared to emergency surgery (37.2%, 16/43), and medical participants (45.1%, 41/91; *p* = 0.003). Drop-out rates varied from 8.3 to 19.5% at 7 days, and 12.5–43.9% at 13 weeks. Age and gender did not differ between patients assessed for eligibility, approached, or recruited. Completion rates were highest for ultrasound quadriceps (98.8%, 80/81 across all groups at baseline). Gait speed completion rates were lower in medical (70.7%, 29/41) compared to elective participants (100%, 24/24) at baseline.

**Conclusion:**

Higher participation refusal and drop-out rates should be expected for research involving recruitment of participants from the acute setting. Assessment of muscle quantity/quality through ultrasound is recommended in early-stage trials in the acute setting, where completion rates of physical performance testing are expected to be lower.

## Key summary points

**Aim:**

To assess the feasibility of conducting acute sarcopenia research in complex populations of hospitalised older adults.

**Findings:**

Recruitment rates were higher in elective surgery patients compared to emergency surgery or medical patients. Drop-out rates were not affected by age or frailty of participants. Completion rates of ultrasound quadriceps were higher than other procedures.

**Message:**

Acute sarcopenia research represents unique challenges but is feasible provided protocol adaptations are incorporated. Assessment of muscle quantity and quality should be included in early-stage clinical research studies to provide mechanistic insights underpinning interventions, especially where physical performance testing may not be possible or reliable.

## Background

Acute sarcopenia is defined by acute reductions in muscle quantity/quality and/or function (strength or physical performance) leading to incident sarcopenia within 6 months, and normally occurs follows a stressor event [1]. It is an increasingly recognised condition in hospitalised patients and older adults are considered particularly vulnerable [2]. Interventional trials are urgently needed to prevent and treat this condition. However, this is an inherently complex population, and trial design needs to be pragmatic to enable clinical translation into the real world [3]. This study presents feasibility data from a prospective observational cohort study of acute sarcopenia, with direct relevance towards trial design for targeted interventions.

## Methods

### Study setting and design

Participants were recruited from the Queen Elizabeth Hospital Birmingham (QEHB) from May 2019 to April 2021. Recruitment was paused between March 2020 and September 2020, and from January 2021 to March 2021 due to the Coronavirus 2019 (COVID-19) pandemic for safety reasons, and to enable redeployment of clinical staff. The full protocol for this study has been published previously [4]. We aimed to involve three cohorts of older patients: elective colorectal surgery, emergency abdominal surgery, and medical patients. Elective patients were recruited from preoperative assessment clinic, with measurements taken prior to admission, within 48 h post-operatively, 7 (± 2) days post-operatively, and 13 (± 1) weeks post-operatively. Emergency surgery patients were recruited from surgical wards preoperatively or post-operatively, with measures taken pre-operatively (if possible), within 48 h post-operatively, 7 (± 2) days post-operatively, and 13 (± 1) weeks post-operatively. Medical patients were recruited from medical wards within 48 h of admission, 7 (± 2) days post-admission, and 13 (± 1) weeks post-admission. Follow-up at 13 weeks took place in the participant’s own home or the Inflammation Research Facility, QEHB. An amendment was added during the COVID-19 pandemic to enable telephone follow-ups at 13 weeks.

### Participant population

All participants were aged 70 years and older and provided written informed consent, or personal or professional consultee declaration was obtained if they were unable to consent for themselves during hospitalisation. If provided written informed consent, additional (optional) consent was obtained for them to remain in the study in the event that they should be unable to consent for themselves during hospitalisation. The elective cohort included patients expected to undergo major colorectal surgery, the emergency surgery cohort included emergency admitted patients who had undergone or were planned to undergo emergency abdominal surgery, and the medical cohort included emergency admitted patients with acute bacterial infections. Following an amendment, patients with symptomatic COVID-19 were also included within the medical cohort [5]. Pre-specified exclusion criteria for all cohorts were inability to understand verbal English, inability to mobilise prior to admission, or life expectancy less than 30 days. Participants were identified by clinicians who were embedded within the direct care clinical team.

## Procedures

### Ultrasound quadriceps

At each visit, rectus Femoris (RF) and Vastus Intermedius (VI) were imaged using B-mode ultrasonography (Venue 50, GE Healthcare) bilaterally and thickness measurements taken not including the fascia, as described previously. Bilateral Anterior Thigh Thickness (BATT) was calculated as the total thickness of all four muscles (right RF + right VI + left RF + left VI) [6].

### Bioelectrical impedance analysis

Bioelectrical Impedance Analysis (BIA) was performed using the Bodystat Quadscan 4000 at each visit. Cardiac devices were considered contraindications to this. Weight and height were used to estimate skeletal muscle mass from resistance and reactance, using previously validated equations [4].

### Handgrip strength

Handgrip strength was measured using a Jamar hydraulic dynamometer by asking the participants to “squeeze as hard as [they] can”. This was measured with the participant sat out with the elbow bent at 90° where possible [7]. Handgrip strength was measured in the bed where participants were unable to sit out in a chair.

### Physical performance

Either usual gait speed alone (four metre course) or Short Physical Performance Battery (SPPB) [8] were measured at each visit (except for the surgical populations within 48 h of surgery).

### Questionnaires

Questionnaires were administered at baseline, 7-day, and 13-week visits including Activities of Daily Living (ADLs–Katz [9], and Lawton [10]), and Patient-Reported Outcome Measures Information System (PROMIS^®^ [11]) Physical Function. Mini-Nutritional Assessment (MNA) Full Form [12] was administered at baseline and 13-week follow-up. An acceptability questionnaire was administered at the final visit.

### Other assessments

Frailty was assessed using a Frailty Index (FI) [13], Clinical Frailty Scale (CFS) [14], and Fried phenotype definition [15], as detailed in the original protocol [4]. Activities of Daily Living (ADLs) were defined by a combined score of Katz (basic) [9] and Lawton (instrumental) [10] ADLs. Common selected morbidities were categorised as binary variables. Delirium was assessed for by the geriatrician researcher and defined according to the Diagnostic and Statistical Manual of Mental Disorders 5 [16]. Source of infection in the medical cohort, and surgical approach in the surgical cohorts were extracted from routinely collected clinical information. Laparoscopic approach includes extended laparoscopic and assisted laparoscopic approaches. Open approach includes laparoscopic surgery converted to open intra-operatively. Other procedures/assessments performed as part of the study included step count using Fitbit Inspire devices (optional), and venepuncture (optional) within 48 h of surgery or admission, and prior to admission in the elective cohort.

### Feasibility outcomes

We recorded numbers of patients who were identified, approached, and recruited for each cohort, and reasons for non-participation. Age and gender were extracted from routinely collected clinical information for patients who were assessed for eligibility and approached to participate but not recruited to the clinical study. Drop-outs and reasons were recorded at each stage. Where it was not possible to perform specific assessments at each visit, this was also recorded. In the case of physical performance testing, if the participant was able to attempt the test but physically unable to complete it, this was considered as completed. However, if the participant declined testing, or it was unsafe or impractical to do so, then this was considered not completed.

### Statistical analysis

The study was originally powered to assess within group differences in PROMIS scores (minimally clinically important difference of 6) from baseline to 13-week follow-up (56 participants in each cohort; 45 to follow-up with 25% drop-out rate) [4]. Due to the study being paused during the COVID-19 pandemic, the recruitment target was revised to enable assessment of differences in PROMIS scores across groups (i.e. minimum of 45 to follow-up across groups). The analysis presented in this manuscript presents the overall feasibility results; a further power calculation was not derived for this analysis. Statistical analysis was performed using IBM SPSS Statistics 26. Baseline characteristics are summarised as means (SD), and frequencies. One-way analysis of variance (ANOVA), Kruskal–Wallis, and Chi-squared tests were used to assess for significance of differences in characteristics between each cohort, and between patients assessed for eligibility, approached, and recruited to the study. Cochran’s *Q* test was used to assess for significance of drop-out rates within groups. Linear mixed models were used to assess for significance of differences in age and FIs within groups. Chi-squared tests were used to assess for significance of drop-out rates between groups, and gender and cognitive disorder differences within and between groups. One-way ANOVA tests were used to assess for significance of differences in age and FIs between groups. One-way ANOVA tests were used to assess for significance of differences in days to follow-up between groups, and chi-squared tests were used to assess for significance of differences in rates of individual assessment completion between groups.

## Results

### Participant characteristics

Table 1 shows the characteristics for all participants across the three cohorts. Eighty-one participants were recruited across all cohorts (24 elective surgery, 16 emergency surgery, 41 medical). The mean age of all participants was 79 years old, and 38.3% (31/81) were females. The majority of participants (93.8%, 75/80) were White British. Mean Body Mass Index (BMI) was 26.7, with no significant difference across cohorts. Participants recruited to the medical cohort were older, with greater risk of being malnourished, higher FIs, higher CFS, lower ADL scores, and greater rates of ischaemic heart disease than the surgical cohorts. There were greater rates of cancer at baseline in the elective cohort, relating to the indication for surgery. The most common source of infection within the medical cohort was respiratory. The majority of operations (85.7%, 12/14) performed within the emergency surgery group were undertaken through an open approach (i.e. emergency laparotomies), which was significantly higher than the emergency surgery group (34.8%, 8/24; *p* = 0.003).

**Table 1:** Baseline characteristics and outcomes of participants

	Overall (*N* = 80)	Elective surgery (*N* = 24)	Emergency surgery (*N* = 15)	Medical (*N* = 41)	*p* value
Baseline characteristics					
Age—mean (SD)	79.2 (6.6)	76.4 (5.3)	75.5 (4.2)	82.1 (6.7)	< 0.001^a^
Gender—Females % (*N*)	38.8 (31)	50.0 (12)	33.3 (5)	34.1 (14)	0.400^b^
Ethnicity % (*N*)					
White British	93.8 (75)	95.8 (23)	100 (15)	90.2 (37)	0.727^b^
White Irish	2.5 (2)	0 (0)	0 (0)	4.9 (2)	
Indian	2.5 (2)	4.2 (1)	0 (0)	2.4 (1)	
Arab	1.3 (1)	0 (0)	0 (0)	2.4 (1)	
Body Mass Index (kg/m^2^)—mean (SD)	26.7 (6.5)	26.4 (4.3)	25.0 (5.0)	27.4 (8.0)	0.472^a^
Nutritional status—% (*N*)					
Normal	42.5 (34)	75.0 (18)	40.0 (6)	24.4 (10)	0.001^b^
At risk	50.0 (40)	25.0 (6)	60.0 (9)	61.0 (25)	
Malnourished	7.5 (6)	0 (0)	0 (0)	14.6 (6)	
Frailty index—mean (SD)	0.27 (0.11)	0.20 (0.09)	0.25 (0.13)	0.32 (0.09)	< 0.001^a^
Clinical Frailty Scale—median (IQR)	4 (3–5)	3 (3–4)	3 (3–4)	5 (4–5)	< 0.001^c^
Katz and Lawton Activities of Daily Living—median (IQR)	13 (11–14)	14 (13–14)	13 (10–14)	12 (10–13)	0.001^c^
Delirium—% (*N*)	15.0 (12)	8.3 (2)	13.3 (2)	19.5 (8)	0.467^b^
Morbidities—% (*N*)					
Diabetes Mellitus	22.5 (18)	12.5 (3)	26.7 (4)	26.8 (11)	0.374^b^
Heart failure	5.0 (4)	0 (0)	0 (0)	9.8 (4)	0.135^b^
Ischaemic Heart Disease	16.3 (12)	0 (0)	20.0 (3)	24.4 (10)	0.033^b^
Stroke	5.0 (4)	0 (0)	0 (0)	9.8 (4)	0.135^b^
Cancer	40.0 (32)	91.7 (22)	33.3 (5)	12.2 (5)	< 0.001^b^
Asthma	12.7 (10)	12.5 (3)	13.3 (2)	12.5 (5)	0.996^b^
Chronic Obstructive Pulmonary Disease	20.0 (16)	20.8 (5)	6.7 (1)	24.4 (10)	0.338^b^
Anxiety/Depression	10.0 (8)	12.2 (5)	6.7 (1)	12.2 (5)	0.787^b^
Pre-existent cognitive impairment	2.5 (2)	0 (0)	0 (0)	4.9 (2)	0.377^b^
Infection source (medical participants only)—%(*N*)					
Respiratory	NA			56.1 (23)	NA
Urinary				9 (22.0)	
Skin				7.3 (3)	
Biliary				2.4 (1)	
COVID-19				7.3 (3)	
Unknown origin				4.9 (2)	
Surgical approach—% (*N*)					
Laparoscopic	45.9 (17)	65.2 (15)	14.3 (2)	NA	0.003^b^
Open	54.1 (20)	34.8 (8)	85.7 (12)		
Outcomes					
Length of stay—median (IQR)	8.5 (5–15)	8 (4–15)	13 (7–20)	8 (5–16.5)	0.177^c^
Length of stay < 5 days—% (*N*)	20.5 (16)	34.8 (8)	6.7 (1)	17.5 (7)	
Inpatient death—% (*N*)	7.5 (6)	8.3 (2)	0 (0)	9.8 (4)	0.463^b^

^a^One-way ANOVA

^b^Chi-squared test

^c^Kruskal–Wallis test

### Screening and recruitment

**Fig. 1:**
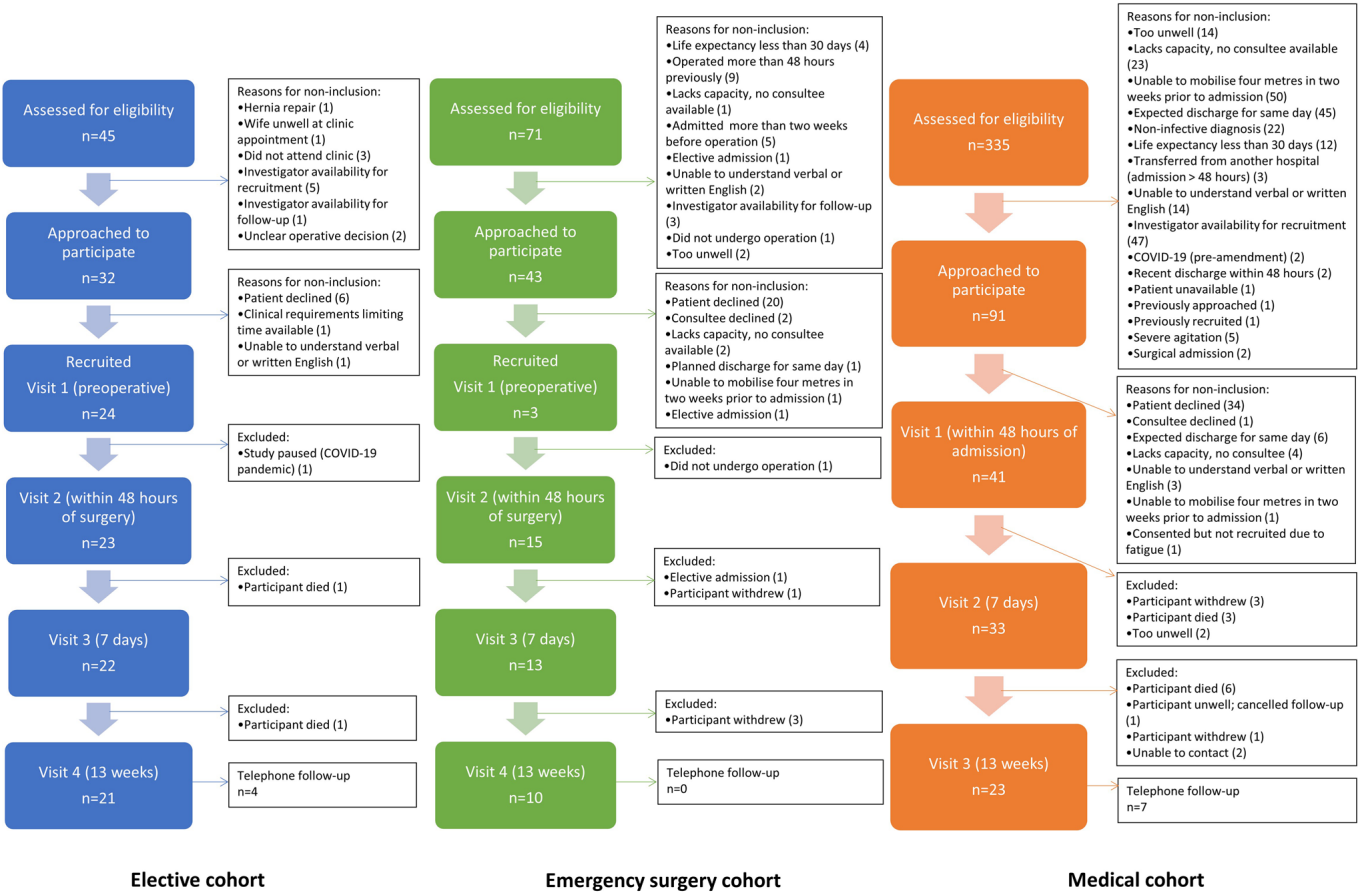
Screening, recruitment, and follow-up rates for participants in all cohorts and reasons for non-participation

Figure 1 shows the recruitment flowcharts for each cohort. Table 2 shows patient/participant demographics for participants screened, approached, recruited, and during follow-up. More participants were identified as potentially eligible in the medical cohort compared to the surgical cohorts. However, percentage of patients assessed for eligibility that were approached was lowest in the medical cohort (27.2%, 91/335 vs 71.1%, 32/45 in elective cohort). The most common reasons for non-inclusion in the medical cohort were the inability to mobilise four metres at baseline, or expected discharge the same day. Although expected length of stay did not form part of the prespecified inclusion/criteria, it was generally considered impractical to recruit patients who were expected to be discharged the same day. The percentage of patients who were approached to participate who were recruited was highest in the elective surgery cohort (75%, 24/32) and lowest in the emergency surgery cohort (37.2%, 16/43; *p* = 0.003). In the emergency surgery cohort, the majority of participants (81.3%, 13/16) were recruited post-operatively. There were no significant differences in age or gender within cohorts between patients assessed for eligibility, approached to participate, and recruited. The only significant difference for the group overall was a higher mean age in patients assessed for eligibility, accounted for by the higher weighting of medical patients within this.

**Table 2:** Screening, recruitment, and follow-up rates for participants separated by cohort and characteristics

	Overall	Elective surgery	Emergency surgery	Medical	p value (across groups)
N numbers					
Screened—*N*	451	45	71	335	
Approached—*N* (% of screened)	166 (26.8%)	32 (71.1%)	43 (60.6%)	91 (27.2%)	< 0.001^a^
Recruited—*N* (% of approached)	81 (48.8%)	24 (75%)	16 (37.2%)	41 (45.1%)	0.003^a^
7-day follow-up—*N* (% of recruited)	67 (82.7%)	22 (91.7%)	13 (81.3%)	33 (80.5%)	0.470^a^
13-week follow-up—*N* (% of recruited)	54 (66.7%)	21 (87.5%)	10 (62.5%)	23 (56.1%)	0.021^a^
*p* value (within group)	< 0.001^b^	< 0.001^b^	< 0.001^b^	< 0.001^b^	
Age—mean (SD)					
Screened	81.2 (7.3)	76.4 (4.8)	76.9 (4.7)	82.8 (7.3)	< 0.001^c^
Approached	79.5 (6.2)	76.7 (5.0)	77.4 (4.6)	81.5 (6.6)	< 0.001^c^
Recruited	79.2 (6.6)	76.4 (5.3)	75.5 (4.2)	82.1 (6.7)	< 0.001^c^
7-day follow-up	78.8 (6.4)	76.1 (4.8)	75.5 (4.2)	81.7 (6.8)	< 0.001^c^
13-week follow-up	78.4 (6.9)	76.0 (4.9)	75.6 (4.2)	82.1 (7.9)	0.004^c^
*p* value (within group)	0.001^d^	0.092^d^	0.425^d^	0.598^d^	
Gender—Females % (N)					
Screened	50.2 (226)	53.3 (24)	49.3 (35)	50.0 (167)	0.902^a^
Approached	48.8 (81)	59.4 (19)	44.2 (19)	47.3 (43)	0.389^a^
Recruited	38.8 (31)	50.0 (12)	33.3 (5)	34.1 (14)	0.400^a^
7-day follow-up	40.6 (28)	54.5 (12)	38.5 (5)	33.3 (11)	0.227^a^
13-week follow-up	44.4 (24)	52.4 (11)	40.0 (4)	39.1 (9)	0.701^a^
*p* value (within group)	0.228^a^	0.968^a^	0.722^a^	0.127^a^	
Baseline Frailty Index—mean (SD)					
Recruited	0.27 (0.11)	0.20 (0.09)	0.25 (0.13)	0.32 (0.09)	< 0.001^c^
7-day follow-up	0.27 (0.11)	0.20 (0.08)	0.25 (0.14)	0.33 (0.08)	< 0.001^c^
13-week follow-up	0.27 (0.11)	0.21 (0.08)	0.23 (0.15)	0.32 (0.09)	< 0.001^c^
*p* value (within group)	0.755^d^	0.989^d^	0.941^d^	0.973^d^	
Cognitive impairment (delirium and pre- existent)—% (*N*)					
Recruited	17.3 (14)	8.3 (2)	12.5 (2)	24.4 (10)	0.218^a^
7-day follow-up	15.9 (11)	4.5 (1)	15.4 (2)	23.5 (8)	0.166^a^
13-week follow-up	13.2 (7)	4.8 (1)	20.0 (2)	18.2 (4)	0.336^a^
*p* value (within group)	0.817^a^	0.830^a^	0.876^a^	0.845^a^	

^a^Chi-squared test

^b^Cochran’s Q tes

^c^One-way ANOVA

^d^Linear mixed models

Considering the reasons why patients who were approached declined to participate, one of the most common reasons was that they felt that they just had “too much going on”; this was frequently cited as a reason for all cohorts. Patients in the surgical cohorts also stated that they wanted to “focus on their operation”. In both the emergency surgery and medical cohorts, many patients also frequently stated that they felt “too exhausted”, “too unwell”, or just “didn’t feel up to it”. One medical patient who was approached expressed quite frankly that they did not want to “be a guinea pig”. Another common reason patients expressed for declining to participate was that, despite assurances, they felt in themselves that they were not appropriate to participate in the research study; “too old”, “mobility not good enough”, “might not be able to complete assessments”, “hearing impairment would make it difficult”.

### Drop-outs and loss to follow-up

Follow-up rates were highest in the elective cohort (7 days: 91.7%, 22/24; 13 weeks: 87.5%, 21/24) and lowest in the medical cohort (7 days: 80.5%, 33/41; 13 weeks: 58.5%, 24/41). These differences were statistically significant at 13 weeks (*p* = 0.032). Participants who chose to withdraw from the study following recruitment cited similar reasons to those who declined initial participation; “too much going on”, didn’t think their data would be “useful to the study”. There were no statistically significant differences in age, gender, baseline FI, or cognitive impairment (both delirium and pre-existent) within groups between patients recruited and included at follow-up. However, there were non-statistically significant lower rates of participants with cognitive impairment at recruitment remaining in the study at follow-up in the medical cohort. More patients died during their inpatient stay in the medical cohort compared to the surgical cohorts, although this also was not statistically significant (Table 1). There was no significant difference in the median length of stay between cohorts. However, in the elective cohort 34.8% (8/24) had a length of stay of less than five days, compared to 6.7% (1/15) in the emergency surgery cohort, and 17.5% (7/41) in the medical cohort. The mean number of days to follow-up from visit 2 (surgical cohorts)/ visit 1 (medical cohort) was 5.5 (SD 1.2) days for 7-day follow-up and 90.8 (SD 7.6) days for 13-week follow-up, and there were no significant differences between groups.

### Feasibility of individual procedures

Table 3 shows the percentage of each assessment completed at each visit for each patient group, accounting for drop-outs and telephone follow-ups. The procedure with the highest completion rates across all visits was ultrasound quadriceps, with only two single occasions when this was not possible in participants who remained in the study. There was one medical participant in whom ultrasound was attempted, but it was not possible to sufficiently delineate the muscle borders due to reduced penetration of sound waves through overlying adipose tissue, and one surgical participant for whom ultrasound was abandoned post-operatively due to agitation. This included completion in a number of different settings, with participant standardised in the position with the upper body semi-upright, and the knees extended in the natural resting position.

**Table 3:** Completion rates of individual procedures separated by cohort and study visit

		Overall	Elective surgery	Emergency surgery	Medical	*p* value
Visit 1/Baseline						
Elective—preoperative assessment clinic Emergency surgery—preoperative (questionnaires may be postoperative) Medical—within 48 hours of admission	Bioelectrical Impedance Analysis	88.2% (60/68)	91.7% (22/24)	100% (3/3)	85.4% (35/41)	0.607
	Ultrasound quadriceps	98.5% (67/68)	100% (24/24)	100% (3/3)	97.6% (40/41)	0.716
	Handgrip strength	100% (68/68)	100% (24/24)	100% (3/3)	100% (41/41)	NA
	Gait speed	81.5% (53/65)	100% (24/24)	NA	70.7% (29/41)	0.003
	Other physical performance tests	83.1% (54/65)	100% (24/24)	NA	73.2% (30/41)	0.005
	PROMIS Physical Function	98.8% (80/81)	100% (24/24)	93.8% (15/16)	100% (41/41)	0.128
	Other questionnaires	98.8% (80/81)	100% (24/24)	93.8% (15/16)	100% (41/41)	0.128
	Venepuncture	75% (51/68)	100% (24/24)	33.3% (1/3)	63.4% (26/41)	0.001
Visit 2 (surgical)						
Elective—within 48 h of surgery Emergency surgery—within 48 h of surgery	Bioelectrical Impedance Analysis	89.5% (34/38)	87.0% (20/23)	93.3% (14/15)	NA	0.531
	Ultrasound quadriceps	97.4% (37/38)	95.7% (22/23)	100% (15/15)	NA	0.413
	Handgrip strength	89.5% (34/38)	87.0% (20/23)	93.3% (14/15)	NA	0.531
	Venepuncture	65.8% (25/38)	60.9% (14/23)	73.3% (11/15)	NA	0.429
Visit 3 (surgical)/Visit 2 (medical)						
Elective—7 (± 2) post-operative Emergency surgery—7 (± 2) days post-operative Medical—7 (± 2) days post-admission	Mean (SD) days from visit 2 (surgery)/ visit 1 (medical)	5.5 (1.2)	5.4 (1.0)	5.0 (1.4)	5.8 (1.1)	0.116
	Bioelectrical Impedance Analysis	87.0% (60/69)	86.4% (19/22)	92.3% (12/13)	85.3% (29/34)	0.811
	Ultrasound quadriceps	100% (67/67)	100% (22/22)	100% (32/32)	100% (13/13)	NA
	Handgrip strength	98.5% (66/67)	95.5% (21/22)	100% (13/13)	100% (32/32)	0.354
	Gait speed	88.1% (59/67)	86.4% (19/22)	92.3% (12/13)	87.5% (28/32)	0.864
	Other physical performance tests	84.4% (27/32)	NA	NA	84.4% (27/32)	NA
	PROMIS Physical Function	94.1% (64/68)	90.9% (20/22)	100% (13/13)	93.9% (31/33)	0.542
	Other questionnaires	98.5% (67/68)	95.5% (21/22)	100% (13/13)	100% (33/33)	0.346
	Fitbit data	51.5% (35/68)	54.5% (12/22)	38.5% (5/13)	54.5% (18/33)	0.580
Visit 4 (surgical)/Visit 3 (medical)						
Elective—13 (± 1) weeks post-operative Emergency surgery—13 (± 1) weeks post-operative Medical—13 (± 1) weeks post-admission	Mean (SD) days from visit 2 (surgery)/ visit 1 (medical)	90.8 (7.6)	89.6 (6.7)	88.7 (2.9)	93.0 (9.3)	0.219
	Bioelectrical Impedance Analysis	90.0% (36/40)	94.1% (16/17)	80.0% (8/10)	92.3% (12/13)	0.470
	Ultrasound quadriceps	100% (40/40)	100% (17/17)	100% (10/10)	100% (13/13)	NA
	Handgrip strength	100% (40/40)	100% (17/17)	100% (10/10)	100% (13/13)	NA
	Gait speed	100% (40/40)	100% (17/17)	100% (10/10)	100% (13/13)	NA
	Other physical performance tests	97.5% (39/40)	100% (17/17)	100% (10/10)	92.3% (12/13)	0.345
	PROMIS Physical Function	98.1% (53/54)	100% (21/21)	100% (10/10)	95.7% (22/23)	0.503
	Other questionnaires	98.1% (53/54)	100% (21/21)	100% (10/10)	95.7% (22/23)	0.532

Completion rates were highest in the elective group at recruitment. A significantly lower proportion of medical participants were able to complete gait speed testing at recruitment compared to elective participants (70.7%, 29/41 vs 100%, 24/24; *p* = 0.003). Completion rates were higher at 13-week follow-up compared to during hospitalisation in all groups, with 100% of ultrasound quadriceps, handgrip strength, and gait speed testing completed in all groups. All elective participants agreed to venepuncture at baseline assessment, compared to 63.4% (26/41) of medical participants (*p* = 0.001). However, rates were lower post-operatively at 60.9% (14/23) in elective participants. Fitbit data during hospitalisation were collected for 51.5% (35/68) of participants across all groups, with no significant difference between groups.

### Capacity, delirium, and cognitive impairment

Consultee declaration was obtained at recruitment in 10% (4/41) of medical participants and 12.5% (2/16) of emergency surgery participants, who were considered to lack capacity at time of recruitment. Consultee declaration was also obtained for an additional medical participant who demonstrated ongoing loss of capacity during the study after initially providing informed consent to participate. Across all cohorts, 97.4% (74/76) of participants provided additional consent to remain in the study in the event that they should be unable to make decisions for themselves during the course of the study. The overall prevalence of delirium in all participants at any point in the study was 15.0% (12/80). This was lowest in the elective cohort (8.3%, 2/24), and highest in the medical cohort (19.5%, 8/41). No participants in the surgical cohorts had pre-existent cognitive impairment. The prevalence of pre-existent cognitive impairment within the medical cohort was 4.9% (2/41).

## Discussion

This study provides important feasibility data on conducting acute sarcopenia research in a complex real-world patient population. The recruitment and drop-out rates demonstrated in this study should be used to guide recruitment targets for future cohort studies and interventional trials. Participation refusal and withdrawal rates were lowest in the elective surgery cohort. This is likely to relate to the recruitment environment within the outpatient department, and the patient’s own clinical stability. However, this cohort was also younger and less frail than the medical cohort. This may also have impacted upon participation rates, although there was no evidence that patients were more likely to drop-out from the study if they were older or more frail.

The reasons that patients and participants expressed for refusal to participate or withdrawal from the study are illuminating. Previous studies have recurrently shown that older adults are under-represented in clinical trials [17]. However, despite reassurances, many patients expressed that they felt they were “too old” for research. In our previous study, we demonstrated that key drivers for research participation amongst older adults were the ability to “give back”, and being able to learn something different [18]. Medical professionals should strive to encourage active participation of older adults in research by engaging with them, and demonstrating how their participation could help other people in the future. Recruitment of participants when clinically stable, and ideally in an outpatient or community setting is encouraged where possible. However, for studies evaluating the acute effects of hospitalisation, this is often not practical. A simplified consent process may assist when patients are especially exhausted from their illness.

Delirium and dementia are common in older adults, and mental capacity may fluctuate throughout the course of hospitalisation [19, 20]. We have shown significant results that, in participants who are able to provide informed consent at recruitment, nearly all would be happy to remain in the study in the event that they were to lose capacity during the course of the study. We consider that all studies involving hospitalised older patients should include this specific consent. In participants who exhibit ongoing loss of capacity during the course of the study, a personal or professional consultee may be consulted in line with the participant’s wishes and national legislation. The overall rates of delirium in this study were similar to the prevalence demonstrated in previous studies in medical and surgical patients [19, 21]. However, the rates of pre-existent cognitive impairment were lower than demonstrated in previous studies [19]. Although this likely partially relates to higher rates of functional impairment (i.e. inability to walk four metres at baseline) in patients with advanced dementia, and higher rates of non-infective reasons for admission (e.g. falls, social concerns) [22], this potentially suggests a bias in recruitment where it was not possible to recruit participants if consultees were unavailable.

The procedure with the overall highest completion rates was ultrasound quadriceps. Ultrasound provides non-invasive real-time assessment of muscle quantity and quality. We previously showed that ultrasound was highly acceptable to participants, associated with low perceived burden when compared to handgrip strength and gait speed testing [18]. Importantly, it was possible to standardise the position that this was performed in in a multitude of settings (outpatient department, inpatient ward, participants’ own homes). Previous research with healthy volunteers has demonstrated that BATT will be affected by concurrent hip and knee flexion, but that small variations in tilt of the upper body can be tolerated so long as the knees are kept in natural extension [23]. However, ultrasound does require more training and expertise than BIA.

Lower completion rates for BIA are entirely accounted for by participants with cardiac devices in-situ. Recently, BIA has been shown to be potentially safe to be performed in participants with cardiac devices, although it is unclear how the presence of cardiac devices may affect the interpretation of the results [24]. It is also important to note that there was one participant in whom it was not technically possible to obtain valid measurements with ultrasound. Gold standard techniques recommended for assessment of muscle quantity are Computed Tomography (CT) and Magnetic Resonance Imaging (MRI) [1]. However, these techniques are not feasible for serial, real-time, or bedside evaluation. We suggest that trials for acute sarcopenia should incorporate both ultrasound and BIA at present, as complementary assessment techniques of muscle quantity. Where possible, gold standard imaging may be performed when stable prior to hospitalisation and at follow-up in studies that aim to explore mechanisms.

Considering the timing of dynamic assessments, the median length of stay across all cohorts was 8.5 days. Therefore, it should be possible to perform repeated measures for most participants, if the first measure is taken within 48 h of admission. However, a third of elective participants, and almost a fifth of medical participants had a length of stay of four days or less. Additionally, a significant number of identified medical participants were not recruited as they were expected to be discharged. Where feasible, repeated measures can be performed in participant’s own homes if they are discharged prior to their planned assessment date; however, this is likely to be impractical and costly for large-scale clinical trials. This may also limit the effectiveness of interventions when these are only delivered to participants during their inpatient stay.

We consider that ultrasound and BIA provide pragmatic tools in demonstrating mechanistic action of effects in interventional trials. These techniques may also demonstrate minimally clinically important difference that might not be demonstrated in other outcomes in preliminary pilot studies. The incorporation of muscle quantity/quality assessment through ultrasound and BIA provides a cost-effective strategy towards demonstrating efficacy in early interventional trials. However, diagnosis of sarcopenia requires demonstration of loss of muscle function, and not just quantity/quality [1]. Completion rates for handgrip strength were higher than physical performance, but it is also recognised that handgrip strength may be affected by fatigue. Trials of interventions for acute sarcopenia should continue to incorporate assessment of muscle function, but the protocols should pre-specify how expected non-completion rates will be accounted for.

It should also be emphasised that patient-reported outcomes should be embedded into any clinical trial design. The PROMIS Physical Function questionnaire is simple to administer and sensitive to change [25]. It is sufficiently broad to avoid ceiling and floor effects. Completion rates at each visit were excellent. Importantly, this questionnaire could be administered over telephone follow-ups when real-time assessment is not possible.

Venepuncture and Fitbit use were listed as optional aspects of this study. This may explain why completion rates are lower for these to procedures. It was possible to obtain additional blood samples for all participants recruited to the elective cohort. This relates to the structure of the preoperative assessment clinic, with the research team embedded within this. Blood tests are performed routinely for all patients in preoperative assessment; therefore, it was possible to obtain additional samples at the same needle puncture. However, during hospitalisation, routine clinical bloods were frequently taken at different times, and, therefore, taking additional blood tests for research would have necessitated additional needle puncture. As well as participant refusal, lower rates of Fitbit usage are likely to be multifactorial. As this was an optional part of the study, the research team may have been less invested in promoting this. Participants were admitted to different locations throughout the hospital, and clinical staff may have been unfamiliar with the devices being used for research. At times devices were lost, particularly between bed moves within hospital.

### Limitations

We recognise that there are a number of limitations to our study. First, we recognise that recruitment rates and drop-out rates may differ in interventional trials. Interventional trials can both positively and negatively affect recruitment, as the perceived potential benefit may be greater, as well as the perceived potential harm. Nevertheless, we consider that the expected identification, recruitment, and drop-out rates demonstrated in our study should guide sample size calculations and recruitment timeframes. Second, our feasibility study itself may be under-powered to demonstrate statistically significant differences in participant characteristics. Previous studies have demonstrated lower recruitment rates amongst females compared to males in early phase clinical studies [26]. Although not statistically significant, a lower percentage of participants recruited to the emergency surgery and medical cohorts were female. Protocols should pre-specify how recruitment technique will be adapted to ensure equal gender representation in research. Lastly, the participants recruited to this study were predominantly White British. The exclusion of participants who were unable to understand verbal or written English may have led to bias towards this population.

## Conclusion

Acute sarcopenia research represents unique challenges. This includes the challenges of recruiting a heterogeneous vulnerable population, and the challenges of recruiting in a complex clinical environment. Completion rates of physical performance tests should be expected to be lower in hospitalised patients compared to completion rates of tests of muscle quantity and quality. Protocols should be carefully and adapted and designed to optimise recruitment, and reduce drop-outs, ensuring that research is acceptable to older adults. Enhancing options for follow-up assessments to include seeing participants in their own homes, and virtually (telephone or video), will assist to reduce drop-out rates. Research participation rates were highest when participants were recruited in the outpatient setting. Embedding observational studies and trial design into ongoing cohort studies may assist with identifying patients and streamlining recruitment.

## Data Availability

The anonymised dataset is available from the corresponding author upon reasonable request.
